# Visual approach computation in feeding hoverflies

**DOI:** 10.1242/jeb.177162

**Published:** 2018-05-22

**Authors:** Malin Thyselius, Paloma T. Gonzalez-Bellido, Trevor J. Wardill, Karin Nordström

**Affiliations:** 1 Department of Neuroscience, Uppsala University, 75124 Uppsala, Sweden; 2 Department of Physiology, Development and Neuroscience, University of Cambridge, Cambridge CB3 2EG, UK; 3 Centre for Neuroscience, Flinders University, GPO Box 2100, Adelaide, SA 5001, Australia

**Keywords:** Retinal size, Approach, Foraging behavior, Looming stimuli, Motion vision, Target detection

## Abstract

On warm sunny days, female hoverflies are often observed feeding from a wide range of wild and cultivated flowers. In doing so, hoverflies serve a vital role as alternative pollinators, and are suggested to be the most important pollinators after bees and bumblebees. Unless the flower hoverflies are feeding from is large, they do not readily share the space with other insects, but instead opt to leave if another insect approaches. We used high-speed videography followed by 3D reconstruction of flight trajectories to quantify how female *Eristalis* hoverflies respond to approaching bees, wasps and two different hoverfly species. We found that, in 94% of the interactions, the occupant female left the flower when approached by another insect. We found that compared with spontaneous take-offs, the occupant hoverfly's escape response was performed at ∼3 times higher speed (spontaneous take-off at 0.2±0.05 m s^−1^ compared with 0.55±0.08 m s^−1^ when approached by another *Eristalis*). The hoverflies tended to take off upward and forward, while taking the incomer's approach angle into account. Intriguingly, we found that, when approached by wasps, the occupant *Eristalis* took off at a higher speed and when the wasp was further away. This suggests that feeding hoverflies may be able to distinguish these predators, demanding impressive visual capabilities. Our results, including quantification of the visual information available before occupant take-off, provide important insight into how freely behaving hoverflies perform escape responses from competitors and predators (e.g. wasps) in the wild.

## Abstract (precis)

**Highlighted Article:** Reconstruction of the take-off and flight of feeding female hoverflies when approached by other insects, and quantification of visual parameters, reveals how freely behaving hoverflies perform escape responses from competitors and predators in the wild.

## INTRODUCTION

Many insects visit flowering plants, serving an important ecological role as pollinators while feeding on pollen and nectar (Gilbert, 1985; Gladis, 1997; Jauker et al., 2012; Kikuchi, 1965; Ssymank et al., 2008). The hoverfly genus *Eristalis*, for example, feeds from flowers during the daylight hours of spring and summer (Howarth and Edmunds, 2000; Ottenheim, 2000). *Eristalis* are Batesian honeybee mimics (Brower and Brower, 1965), probably as a defense against predatory birds, with a similar foraging pattern to that of bees in terms of flight velocity, distance and flight time between visited flowers (Golding and Edmunds, 2000; Golding et al., 2001). The *Eristalis* genus is found across the world, including the Himalayas (Shah et al., 2014), Australia (Hull, 1937) and Europe (Francuski et al., 2013).

Female *Eristalis* hoverflies are often found close to the flowers from which they feed (Gilbert, 1981, 1985), often in the presence of other insects (Golding and Edmunds, 2000; Rashed and Sherratt, 2007). When feeding from large flowers, such as sunflowers, hoverflies may feed together with other insects (Kikuchi, 1962a, 1963). However, if feeding from smaller flowers, such as daisies, the occupant hoverfly often evades approaching insects (Kikuchi, 1962b), in many cases leading to neither of the two insects staying on the flower. Whereas some insects approaching the flower may compete for food, others, such as wasps, pose a survival risk (Akre, 1982). Indeed, wasps have been shown to actively predate on different insects, including hoverflies (Harris and Oliver, 1993; Rashed and Sherratt, 2007; Richter, 2000). For the occupant hoverfly, there is thus a trade-off between staying, which poses a risk of getting eaten or injured, and leaving the flower, which leads to lost feeding time and energy intake (Cooper and Frederick, 2007).

When animals flee from a potential threat, the flight direction is most often directed 90–180 deg away from the threatening stimulus, although it also depends on factors such as morphological constraints and the potential presence of a refuge (Domenici et al., 2011; Ilany and Eilam, 2008; Kaiser et al., 1992). Such escape responses may be triggered by a range of visual factors. For example, in houseflies, escape responses appear to be triggered by the increasing contrast of an expanding stimulus (Holmqvist and Srinivasan, 1991), whereas fruit flies and locusts initiate escape responses to looming stimuli 50 ms after the angular size reaches a threshold 50–60 deg (Fotowat et al., 2009; Fotowat and Gabbiani, 2007). In laboratory experiments, the escape response in the crab *Neohelice granulate* is based on the angular increment of the looming stimulus, i.e. how fast its angular size grows on the retina (Oliva and Tomsic, 2012), whereas fiddler crabs observed in the field use a mixture of elevation, size and angular speed (Hemmi, 2005).

The visual optics of both male and female *Eristalis* have dorsofrontal interommatidial angles around 1 deg and a region of binocular overlap (Straw et al., 2006). *Eristalis* photoreceptors (Horridge et al., 1975) show sensitivity across a broad part of the wavelength spectrum. Higher-order interneurons in the third optic ganglion provide sensitivity to optic flow motion (Nordström et al., 2008), similar to what has been found in many other insects (Borst, 2014). Recent work on the hoverfly *Episyrphus balteatus* (Goulard et al., 2015, 2016) showed that they have exquisite optomotor behaviors, likely supported by these neurons (Borst, 2014; Nordström et al., 2008). Furthermore, *Eristalis* hoverflies have neurons specifically tuned to the motion of objects that move relative to the remaining surround – such is the type of motion that would be generated by another insect flying in the vicinity (Nordström, 2012). However, with the exception of the classic studies in the 1970s (Collett and King, 1975; Collett and Land, 1975, 1978), hoverfly target tracking behaviors have been relatively poorly described. Thus, although the hoverfly visual system is relatively well studied, their natural behaviors remain more poorly understood.

To increase our understanding about the natural visually guided behavior in hoverflies, we quantified the escape response of female *Eristalis* feeding from flowers in the field. We found that 94% of occupant females left the flower from which they were feeding when approached by another insect. Even if the incomer did not appear to perform an active attack, the occupant appeared to perform an active escape response, leaving the flower at ∼3 times higher speed when approached by another insect, compared with spontaneous take-offs. We found that the hoverflies took-off upward and forward, and that the direction of take-off depended on the incomer's approach angle. We also found that the angular velocity or the angular increment of the incomer (often referred to as tau) may trigger occupant take-off, as these were similar for approaches by different species. In addition, female hoverflies left the flower from which they were feeding sooner, and at a higher speed, if the incomer was a wasp, suggesting the hoverflies distinguish these predators, at least at the behavioral level.

## MATERIALS AND METHODS

### Recordings and definitions

**Fig. 1.**
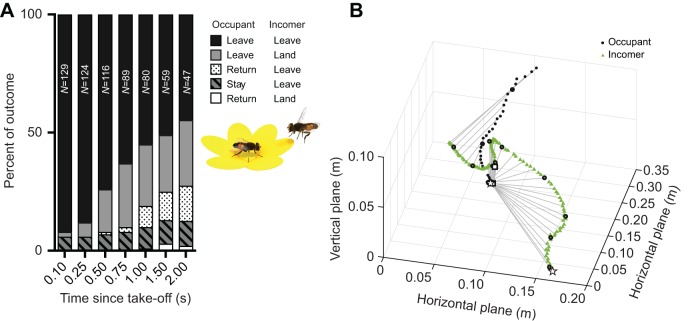
**Interaction outcome.** (A) The bar graph shows the outcome of interactions between a feeding female *Eristalis* (occupant) and an approaching insect (incomer). We defined behaviors as ‘leave’, when an insect left the flower, ‘return’, when the occupant returned to the flower after take-off, ‘stay’, when the occupant remained on the flower, or ‘land’, when the incomer landed on the flower (see color coding and inset). We followed each interaction for as long as possible, where *N* shows the number of interactions analyzed for each time point. (B) Example interaction between two female *Eristalis*, occupant and incomer, where both left the flower. The gray lines connect the path of the occupant and incomer every 25 ms. A black circle marks every 100 ms. White squares indicate time of take-off. White stars indicate the start position of the occupant and incomer. The interaction can be viewed in 3D in Movie 1.

**Fig. 2.**
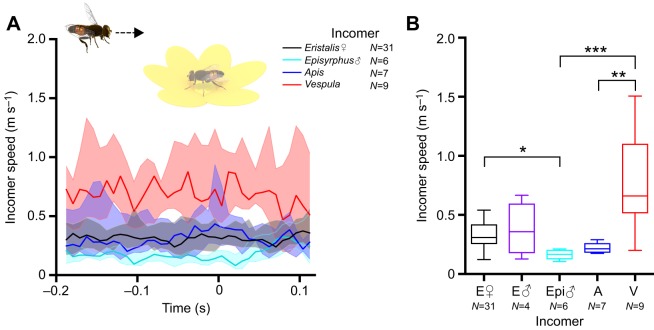
**Incomer speed does not increase as it approaches the flower.** (A) Incomer speed as a function of the time of occupant take-off. The color coding indicates incomer identity; time *t*=0 is the last frame before the occupant took off from the flower. Thick lines show median, shadowing shows the interquartile range. The data have been smoothed with a third-order Butterworth filter with a cut-off frequency of 0.5. We checked for outliers (Tukey) every 50 ms and excluded any insect that was classified as an outlier for a minimum of 4 time points. (B) Box plot of mean incomer speed as measured over 83 ms (10 frames), 100 ms before occupant take-off. E, *Eristalis*; Epi, *Episyrphus balteatus*; A, *Apis mellifera*; V, *Vespula*. The midline is the median and error bars are after Tukey. Statistical significance was tested using one-way ANOVA: *P<0.05, **P<0.01, ***P<0.001.

The natural behavior of hoverflies of the genus *Eristalis*, *Episyrphus balteatus* hoverflies, *Vespula* wasps and *Apis mellifera* honeybees was recorded in Uppsala, Sweden (59°51′N/17°37′E), during July–September 2015 on sunny, calm days, between 10:00 h and 17:00 h. Hoverfly sex was identified visually, using the sexually dimorphic eyes (Collett and Land, 1975), behavior (Heal, 1987; Wijngaard, 2013) or abdominal coloration (Heal, 1979, 1981). All *Eristalis* incomers were female, except for 6 shown in Fig. 1A, and 5 shown in Fig. 2B; all *E.* *balteatus* incomers were male, except 4 in Fig. 1A. The sex of bees and wasps was undetermined.

For videography, two high-speed cameras (120 frames s^−1^ with a resolution of 640×480 pixels; EXFH25, Casio, Tokyo, Japan) were placed on tripods (Dörr cybrit medi 4-BA, Dörr GmbH, Neu-Ulm, Germany; SIRUI T-2005X, SIRUI, Verona, NJ, USA). The cameras were synchronized with a 1 frame resolution using the flashlight of a mobile phone (iPhone 4S, Apple Inc., Cupertino, CA, USA). During recordings, we took audio notes (Voice memos, Apple Inc.) of sex and genus. Care was taken to avoid the experimenter casting shadows on the insects.

The ‘occupant’ was characterized as the insect on the flower at the start of the interaction and the ‘incomer’ as the approaching insect (using the terminology of Kikuchi, 1962b). ‘Take-off’ was defined as the occupant flying away from the flower, with time *t*=0 as the last frame before take-off. ‘Leave’ was defined as the insect leaving the flower. ‘Return’ was defined as the occupant landing on the flower after take-off. ‘Stay’ was defined as those occasions when the occupant did not leave the flower despite an incomer either landing on the flower or on the occupant itself. ‘Land’ was defined as the incomer landing on the flower after occupant take-off. Each interaction was followed for as long as the two individuals were in camera view, or until they had moved on to other interactions or behaviors (e.g. landing on another flower).

### Tracking and 3D reconstruction

3D reconstructions were carried out using custom-written Matlab (MathWorks, Natick, MA, USA) scripts (modified from Wardill et al., 2017). For calibration, we used a 7×7 square checker pattern printed on white paper and glued to a piece of cardboard (as in Wardill et al., 2017). Four sizes of squares were used, with sides of 8.5, 16.6, 21 and 35 mm, respectively. A new calibration was made each time the cameras were moved. The checker pattern was moved horizontally and vertically, for long enough to attain at least 600–1200 frames with the entire pattern clearly visible from both cameras. These frames were then converted to a calibration file using custom-written Matlab scripts (modified from Wardill et al., 2017). For the generation of the calibration file, we used a minimum of 50 frames of the smallest resolved pattern size. For synchronization of the two cameras, we used the synchronization flash to align their frames manually. On a few occasions when the synchronization flash was not visible in both cameras, another distinguishable feature in the videos, such as a rapid flick of a flower petal, was used for synchronization. Synchronizations were then manually verified for at least 10 consecutive frames.

We tracked the position of each insect to get its *x*–*y* coordinates as seen by each camera, i.e. its 2D position. Often, tracking of insect positions had to be done manually, as the contrast against the cluttered background was too low for the process to be reliably automated. In all cases, the center of mass of each insect was used as its position in each frame of each camera. We next calculated the 3D position of each insect in each frame using the calibration file and the 2D location of each insect from each camera (Wardill et al., 2017).

### Quantification of parameters

The 3D distance between the incomer and occupant was calculated using the formula for Euclidian distance (Eqn 1):
(1)
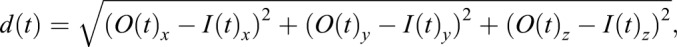
where *d* is distance, *t* is time, *I* is the 3D coordinates of the incomer, *O* is the 3D coordinates of the occupant, and *x*, *y* and *z* are the 3D elements of the 3D coordinates.

**Fig. 3.**
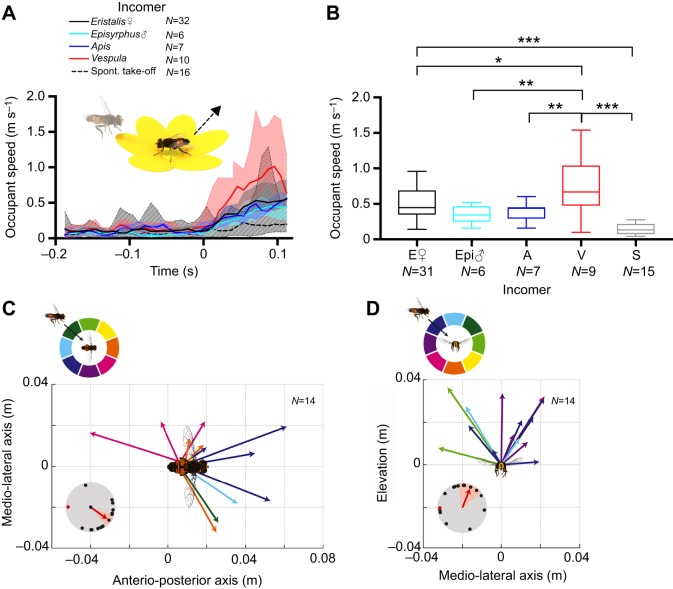
**The occupant performs an escape response.** (A) Occupant speed, color coded according to incomer identity (see key), as a function of the time of occupant take-off (at *t*=0). The dashed line shows the take-off speed when the occupant left the flower spontaneously. Thick lines show median, shadowing shows the interquartile range. The data have been smoothed using a third-order Butterworth filter with a cut-off frequency of 0.5. *t*=0 is the last frame before the occupant took off from the flower. We checked for outliers (Tukey) every 50 ms and excluded any insect that was classified as an outlier for a minimum of 4 time points. A two-way ANOVA with Tukey's multiple comparisons test from *t*=0 to 100 ms showed a time effect (*P*<0.001), species effect (*P*<0.001) and subject effect (*P*<0.001). (B) Box plot of occupant speed 50 ms after occupant take-off. E, *Eristalis*; Epi, *Episyrphus balteatus*; A, *Apis mellifera*; V, *Vespula*; S, spontaneous take-off. The midline is the median and error bars are after Tukey. Statistical significance was tested using one-way ANOVA followed by Tukey's multiple comparisons test: *P<0.05, **P<0.01, ***P<0.001. (C) The arrows show the positions of 14 occupants (female *Eristalis*) 100 ms after take-off, aligned to the position of the occupant at *t*=0, when viewed dorsally (as illustrated in the pictogram). The arrows are color coded to indicate the incomer approach angle (see color coding above graph). The inset shows occupant take-off angle (black dots) as a function of incomer position (red dot), where the red arrow indicates the mean (±s.e.m.) take-off angle. (D) The arrows show the positions of the same 14 occupants (female *Eristalis*) 100 ms after take-off, aligned to the position of the occupant at *t*=0, when viewed anteriorly (as illustrated in the pictogram). The arrows are color coded to indicate the incomer approach angle (see color coding above graph). The inset shows occupant take-off angle (black dots) as a function of incomer position (red dot), where the red arrow indicates the mean (±s.e.m.) take-off angle.

The instantaneous speed for the occupant (Fig. 3A) or incomer (Fig. 2A) was calculated using the Euclidian 3D distance between two consecutive frames (Eqn 2):
(2)
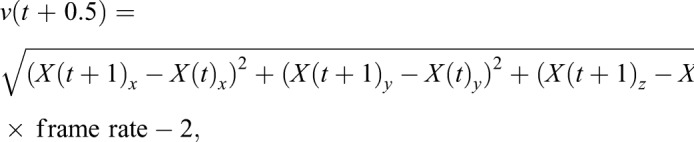
where *v*(*t*) is the speed relative to the ground, *t* is time, *X* is the 3D coordinates of the insect, and *x*, *y* and *z* are the 3D elements of the 3D coordinates. To correct for slight imperfections in the automated tracking, a speed of 2 cm s^−1^ was removed based on tracking a stationary object. The mean incomer speed was calculated by averaging across 10 frames, 100 ms before occupant take-off.

When calculating the retinal size, θ, of the incomer, we used either its width or its length (both *w*) to provide the range of possible angular sizes:
(3)
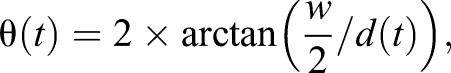
where the width is 0.2 cm for *E*. *balteatus* and 0.4 cm for the other incomers, and the length is 1.0 cm for *E*. *balteatus* and 1.2 cm for the other incomers.

The angular velocity, φ, of the incomer as projected on the occupant's retina was calculated using the law of cosine followed by multiplication by the camera frame rate:
(4)
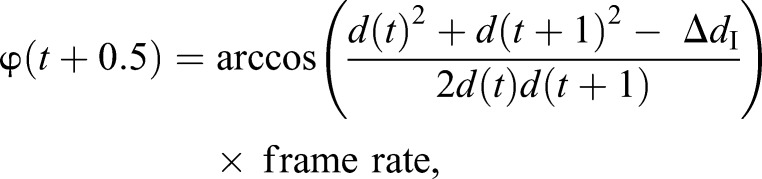
where Δ*d*_I_ is the distance the incomer travelled between time *t* and *t*+1.

Angular increment, τ, was calculated from the retinal size (Eqn 3):
(5)
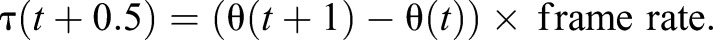


The take-off angle was calculated by identifying the top of the head and the tip of the abdomen of the occupant at the time of take-off, the position of the incomer 100 ms before take-off, and the position of the occupant 100 ms after take-off. We used the body orientation of the occupant just before take-off to normalize the data from the different animals and interactions. For this, we translated the four positions so that the tip of the occupant's abdomen was located at the origin (0,0,0). Next, we rotated the matrix so that the occupant's body was positioned along the positive *x*-axis at take-off.

### Statistics

Prism (Prism 7, GraphPad Software Inc., La Jolla, CA, USA) was used for statistical analysis. We removed statistical outliers, which were classified after Tukey (1993). For analysis of significance, we first performed a D'Agostino and Pearson omnibus normality test, followed by a Kruskal–Wallis test with Dunn's *post hoc* test for non-parametric data, and two-way ANOVA for parametric data. Three levels of significance were used, with *P*<0.05, *P*<0.01 and *P*<0.001 denoted with 1, 2 or 3 asterisks, respectively. Where we show changes over time, we indicate median and interquartile ranges. The whiskers in the boxplots use the Tukey setting in Prism.

## RESULTS

### Female *Eristalis* leave their food flowers when approached by another insect

To quantify the reaction of female *Eristalis* feeding from flowers when approached by incoming insects, we filmed natural interactions during calm sunny days. In this study, none of the flower species (Table 1) from which the female *Eristalis* were feeding had large corollas, and therefore we hypothesized that when approached by other insects, the occupant hoverfly would perform an evasive maneuver (Kikuchi, 1962b, 1963). Indeed, when approached by other hoverflies, bees or wasps (Table 1), the occupant female *Eristalis* left the flower in 94% of the interactions (black and gray data, Fig. 1A).

**Table 1.**
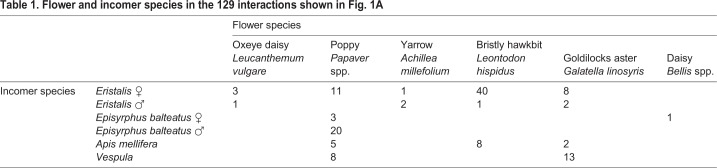
**Flower and incomer species in the 129 interactions shown in** **Fig. 1****A**

Time of take-off was defined as the last frame before the occupant left the flower. Our data show that 100 ms after occupant take-off, only 3 incomers (2.3%; Fig. 1A, gray) had landed on the flower. The incomer return rate increased over time and stabilized at ca. 26% after 750 ms (Fig. 1A, gray). The occupants that left the flower and subsequently returned started doing so only after 500 ms (Fig. 1A, dotted), with the return rate increasing over time and stabilizing at ca. 12% after 1.5 s (Fig. 1A, white and dotted). In the majority of the interactions (55%, Fig. 1A, black), the flower was still vacant 1 s after occupant take-off. Fig. 1B shows a 3D reconstruction of such an interaction, where a feeding occupant female *Eristalis* (black circles) was approached by another female *Eristalis* (green triangles), resulting in both leaving the flower (Movie 1 provides an animation of the same data).

### The incoming insect does not increase its speed on approach

When an insect approaches a flower, it is possible that it is unaware of the occupant hoverfly, perceives it as irrelevant or, alternatively, that it actively attacks the occupant to gain residency of the flower. As several species have been shown to accelerate while approaching a target (Boeddeker et al., 2003; Collett and Land, 1975, 1978), we hypothesized that a directed attack could be associated with an increase in speed as the incomer approached the occupant. To investigate this, we quantified the speed of hoverflies, bees and wasps approaching an occupant female *Eristalis*, from 200 ms before to 100 ms after occupant take-off (Fig. 2A). This analysis showed that the incomer speed was constant over time (species effect: *P*<0.001, subject effect: *P*<0.001, time effect: not significant, two-way ANOVA with Tukey's multiple comparisons test; Fig. 2A), indicating that the incomer was unlikely to be actively attacking the occupant. However, note that wasps flew significantly faster than the other insects during the entire interaction (Fig. 2A, red), which was confirmed by quantifying the mean speed over 10 frames, 100 ms before occupant take-off (one-way ANOVA; Fig. 2B). Note also that *Eristalis* females flew faster than *E*. *balteatus* males (one-way ANOVA; Fig. 2B).

### The occupant hoverfly performs a directed take-off

We next investigated how female *Eristalis* left the flower when approached by an incoming insect, and compared this with spontaneous take-offs, i.e. those that were not induced by an incomer. We found that female *Eristalis* flew away from the flower at a higher speed when approached by an incoming insect (Fig. 3A, colored lines) than when they left the flower spontaneously (Fig. 3A, dashed line). At 50 ms after take-off, the speed was 0.15±0.02 m s^−1^ (mean±s.e.m.) when spontaneously leaving the flower (Fig. 3B, gray), compared with 0.76±0.14 m s^−1^ if the incomer was a wasp (Fig. 3B, red) or 0.52±0.04 m s^−1^ if the incomer was another *Eristalis* female (Fig. 3B, black). In comparison, female *Eristalis* flying between flowers traveled at a speed of 0.34±0.02 m s^−1^ (Fig. 2A, black).

The finding that the occupant left the flower at a higher speed when approached by another insect than when the take-off was spontaneous (Fig. 3A,B) suggests that the occupant could be performing an escape response. If so, we would expect the take-off angle to be consistently directed away from the incomer (Domenici et al., 2011). To investigate this, we determined the occupant's location 100 ms after take-off and aligned the data to the body orientation and position of the occupant in the frame before take-off (at *t*=0, Fig. 3C; Fig. S1A). We plotted the occupant's position as a vector, which was color coded according to the approach angle of the incomer. The data from 14 interactions between two female *Eristalis* show that the take-off angles were directed forward (11 out of 14 take-offs; Fig. 3C) and upward (14 out of 14 take-offs; Fig. 3D; Fig. S1B), suggesting biomechanical limitations or flight direction preferences. In addition, the data indicate that if the incomer came from the right, the occupant tended to fly to the left (Fig. 3C, pink and blue vectors; Fig. 3D, purple and blue vectors), suggesting that the occupant could determine the approach angle of the incomer.

To determine whether occupant take-off was indeed directed away from the incomer, we measured the occupant's take-off angle relative to the approach angle of the incomer. The red dot in the inset in Fig. 3C illustrates the approach angle of the incomer 100 ms before occupant take-off, and the black dots show the resulting take-off angle of the occupant, measured 100 ms after take-off. We found that in azimuth, the average take-off angle was directed 215±15 deg away from the incomer, suggesting that the occupant could determine the incomer's approach angle. The take-offs showed a larger variation in elevation, directed 110±30 deg away from the incomer (Fig. 3D, inset). Considering that female *Eristalis* could not fly below the flower (Fig. 3D), and that they tended to fly forward (Fig. 3C), these take-offs were likely efficient for the occupant hoverfly (Fig. 3C,D, insets), supporting our hypothesis that the take-off was an active escape response away from the intruder (Domenici et al., 2011).

### What visual information is available before take-off?

**Fig. 4.**
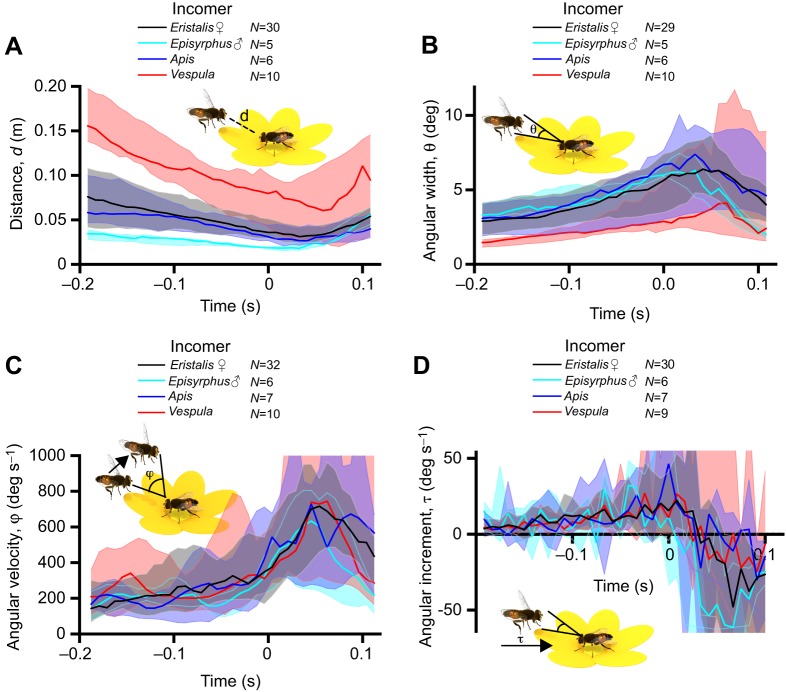
**Visual parameters available to the occupant.** (A) The distance, *d*, between the occupant and the incomer. (B) The angular width, θ, of the incomer as seen by the occupant. (C) The angular velocity, φ, of the incomer as seen by the occupant. The data have been smoothed using a third-order Butterworth filter with a cut-off frequency of 0.25. (D) The angular increment, τ, of the incomer as seen by the occupant. The data have been smoothed using a third-order Butterworth filter with a cut-off frequency of 0.25. In all panels, the color coding indicates incomer identity, *t*=0 is the last frame before the occupant took off from the flower, thick lines show median and shadowing shows the interquartile range. In all panels, we checked for outliers (Tukey) every 50 ms and excluded any insect that was classified as an outlier for a minimum of 4 time points.

What cues might the occupant *Eristalis* hoverfly use for determining when to leave the flower from which it was feeding? Take-off could be triggered by a visual threshold, such as the distance to the incomer, the retinal size or angular velocity of the incomer and its angular increment (e.g. Fotowat et al., 2009; Fotowat and Gabbiani, 2007; Hemmi, 2005; Nityananda et al., 2016; Olberg et al., 2005; Oliva and Tomsic, 2012), or it could be initiated by internal factors that we did not measure here. If a fixed visual variable determines occupant take-off, it should show low variation across interactions (Fotowat and Gabbiani, 2007; Holmqvist and Srinivasan, 1991). To investigate this, we first calculated the distance, *d*, between the occupant and the incomer as a function of time, and found that the distance varied significantly and substantially between the approaches (Fig. 4A). Indeed, whereas the distance to approaching bees (Fig. 4A, dark blue) and other female *Eristalis* (Fig. 4A, black) at take-off was quite similar, the occupant left the flower when wasps were further away (Fig. 4A, red) and when *E*. *balteatus* males were closer (Fig. 4A, turquoise). The differences in distance associated with approaches by particular insects (effect of time, species, subject and interaction, *P*<0.001, two-way ANOVA from *t*=−200 ms to *t*=0) therefore argues against physical distance being a reliable trigger for occupant take-off.

We next quantified the angular size, θ, of the incomer as seen by the occupant, and used the width of the approaching insect as a conservative estimate of its size (see Fig. S2 for corresponding data for incomer length). We found that, compared with the other incomers, the approaching wasps projected a smaller angular size, θ, on the occupant's retina, but the difference was not significant (effect of time: *P*<0.001, effect of subject: *P*<0.001, no effect of species or interaction, two-way ANOVA from *t*=−200 ms to *t*=0; Fig. 4B). Nevertheless, as there was a large variation in the incomer's angular size between approaches (Fig. 4B, shaded areas) over the time leading up to occupant take-off, we found it unlikely that angular size could be the sole determinant of the time of take-off of the feeding female *Eristalis*.

We next quantified the angular velocity, φ, of the incomer as seen by the occupant, and found that the median trajectories of the different approaching species overlaid each other (effect of time: *P*<0.001, effect of subject: *P*<0.001, no effect of species or interaction, two-way ANOVA from *t*=−200 ms to *t*=0; Fig. 4C). We additionally quantified the angular increment, τ, i.e. how fast the image of the incomer grew on the occupant's retina, and found that the median trajectories of the different approaching species overlaid each other (effect of time: *P*<0.001, effect of subject: *P*<0.05, no effect of species or interaction, two-way ANOVA from *t*=−200 ms to *t*=0; Fig. 4D). However, for both these variables, the variation across trials was larger (Fig. 4C,D, shaded areas) than would be expected if the parameter served a role as a threshold trigger. Taken together, the data in Fig. 4 suggest that female *Eristalis* could potentially use a decision filter based on the incomer's angular velocity (φ) or angular increment (τ) for deciding when to take off from the flower on which they were feeding, but that other internal factors such as perceived risk, hunger or attention (Cooper and Frederick, 2007; Dukas, 2001; Ydenberg and Dill, 1986) likely influenced the decision, as the variation across interactions was large (Fig. 4C,D).

## DISCUSSION

In the summer time, hoverflies are often seen interacting among flowers in gardens and fields. Importantly, as they do not readily share small flowers with other insects (Kikuchi, 1962b), when approached by another insect they need to determine whether and when to leave. We showed here that 94% of feeding female *Eristalis* left the flowers on which they were feeding when approached by other insects (Fig. 1). The approaching insect did not appear to perform an active attack against the occupant (Fig. 2), but nonetheless, the occupant left the flower with a fast escape response away from the incomer (Fig. 3). Finally, we also showed that a fixed visual threshold does not explain the timing of the female *Eristalis* take-off, but that the incomer's angular velocity (φ) or angular increment (τ) could play a role in the decision (Fig. 4).

### Interactions on flowers

*Eristalis* hoverflies are Batesian bee mimics, probably as a defense against predators (Brower and Brower, 1965). Indeed, frogs that have been stung by honey bees eat fewer *Eristalis* than frogs that have not experienced a bee sting (Brower and Brower, 1962), and naive human subjects frequently confuse *Eristalis* hoverflies with honeybees (Golding et al., 2005a). Hoverflies show similar flight patterns to bees when foraging around flowers, where they both fly in small loops around the flowers when foraging, as opposed to muscid flies that tend to fly in straight lines between the flowers (Golding and Edmunds, 2000; Golding et al., 2001). The cruising speeds of foraging *Eristalis tenax* and bees (*Apis mellifera*) are also similar (approximately 0.2 m s^−1^; Fig. 2B; see Golding et al., 2005b; Golding et al., 2001). *Vespula vulgaris* wasps have previously been described to fly between flowers at speeds of ca. 0.15 m s^−1^ (Golding et al., 2005b, 2001), or at 0.2 m s^−1^ in a wind tunnel (Brown et al., 2013), which is slower than our data (Fig. 2B, red), but this could depend on factors such as local temperature and time of day.

Hoverflies have previously been shown to not share the flowers from which they are feeding (Kikuchi, 1962a), unless these are large (Kikuchi, 1963), which was confirmed in our study (Fig. 1A). Male hoverflies are highly territorial and similarly avoid sharing their hovering territory with other hoverflies (Fitzpatrick, 1981). However, whereas male *Eristalis* hoverflies readily pursue other insects, including bees, butterflies and even wasps or hornets, sometimes with a lethal outcome for the hoverfly (Fitzpatrick, 1981; Fitzpatrick and Wellington, 1983), female *Eristalis* do not perform such high-speed pursuits (Collett and Land, 1975; Fitzpatrick, 1981). Furthermore, as the flight velocity of the male *Eristalis* and *E*. *balteatus* was low (Fig. 2), the interactions that we filmed here are quite different from high-speed territorial pursuits. Taken together, we find it unlikely that the female behaviors that we described here are territorial; rather, they illustrate a trade-off between exploiting a food source (Cooper and Frederick, 2007), which guarantees food and poses a risk of getting injured, and leaving, which is a safer option that results in immediate energy expenditure and a loss of food intake.

Such trade-offs are important as the approaching insect could be a predator, e.g. a wasp, whose prey range includes hoverflies (Harris and Oliver, 1993; Richter, 2000). Even if wasps are of similar size to bees and other *Eristalis* hoverflies, they fly faster (Fig. 2), and this information could potentially be used by the occupant *Eristalis* (but note that there was no difference in the resulting angular velocity as perceived by the occupant; Fig. 4C). We found that when the incomer was a wasp, the occupant *Eristalis* hoverfly left the flower significantly sooner (i.e. when the wasp was further away, at 0.11±0.014 m compared with 0.026–0.057 m for the other incomer species; Fig. 4A) and its take-off speed was higher (0.62±0.085 m s^−1^ compared with that when approached by other insect species of 0.26–0.40 m s^−1^; Fig. 3A). As the occupant speed 50 ms after take-off was significantly higher in response to wasps than in response to other insects (Fig. 3A), this suggests that the feeding hoverfly might perceive the level of threat posed by a wasp, maybe by using the combined information provided by its higher speed (Fig. 2A) and other visual cues. Indeed, at the time of occupant take-off, the width of the wasp subtends a few degrees on the retina (Fig. 4B), which is larger than the optical resolution of the female hoverfly eye (Straw et al., 2006), and might thus be enough for its unique features to be identified, especially taking into account that dipteran hyperacuity may be 4 times better than predicted by the optics alone (Juusola et al., 2017). Wasps are so different from bees and hoverflies that pigeons can be trained to separate them based on antenna length, contrast of abdominal patterns and number of stripes (Bain et al., 2007; Dittrich et al., 1993). However, not all species are deterred by the markings, as recent work suggests that dragonflies are not discouraged by the classic black/yellow warning signals, such as those of wasps, when pursuing artificial prey (Duong et al., 2017). This could obviously depend on other factors, such as a much thicker cuticle, and the large size difference between dragonflies and their prey.

### Escapes and attacks

We found that the approach by the incoming insect was unlikely to be an active attack, as we saw no difference in flight speed leading up to the interaction (Fig. 2A). Instead, it was more likely that the approaching insects were focused on foraging. Indeed, even if flowers of the same species look quite similar to the human observer, a combination of scent, color and shape make some much more attractive than others (Nordström et al., 2017), which could make a potential food source more salient than the presence of an occupant. It has been suggested that hoverflies might partially base their flower preference on the morphology of their mouth parts (Gilbert, 1980, 1981), whereas others argue that hoverflies rarely display a strong flower preference, but instead visit the most abundant flower in their surroundings (Branquart and Hemptinne, 2000). Nevertheless, both hoverflies and bees readily feed on nectar and pollen from a large variety of flowers (Gilbert, 1985; Nordström et al., 2017; Wallace et al., 2002).

We found that the approaching insect was unlikely to perform a directed attack. In contrast, we found it likely that the occupant performed an escape response as the occupants left the flower at higher speed if they were approached by another insect than if they left the flower apparently spontaneously (Fig. 3A,B). In addition, when approached by other insects, the occupants left the flower faster (Fig. 3A,B) than they approached it (Fig. 2B, black). Previous work, which did not separate spontaneous take-offs from those triggered by an incoming insect, also found that the take-off speed was faster than the approach speed. Such studies interpreted the faster take-off as a general strategy to avoid predators (Golding et al., 2001). By separating spontaneous take-offs from those triggered by an incomer, we found that the fastest take-offs were indeed triggered by the most dangerous incomer, the predatory wasp (Fig. 3A,B, red). We also showed that the escape response was directed 215 deg away from the incomer in azimuth (Fig. 3C, inset) and 110 deg away from the incomer in elevation (Fig. 3D, inset). Mice flee from flying predators at an angle of 45–135 deg away from the incoming threat (Ilany and Eilam, 2008), and mysid crustaceans flee from their predators at a 90 deg angle (Kaiser et al., 1992). In *Drosophila*, the take-off angle is roughly 180 deg if an artificial looming stimulus comes directly from the back or the front, but approximately 90 deg when the stimulus comes from the side (Card and Dickinson, 2008).

Our results (Fig. 3C,D) thus suggest that the hoverflies were able to take the approach angle of the incomer into account, even if they preferred flying forward and upward. Female hoverflies have neurons tuned to the motion of small targets (Nordström and O'Carroll, 2006), which would be suitable for detecting passing insects. These neurons tend to have very large receptive fields, covering a large portion of the ipsilateral or contralateral visual field (Nordström and O'Carroll, 2006). Such neurons would thus alert the hoverfly to the presence of a small target moving across the visual field, but not provide more detailed position or direction information. Furthermore, as the incomer gets closer to the occupant, it would become a looming stimulus. The neural network of looming-sensitive neurons, underlying fly escape responses, have been described in amazing detail in *Drosophila* (von Reyn et al., 2017).

### Variables triggering take-off

If a certain visual parameter serves as a threshold trigger of take-off, it should have small variance at a fixed time before take-off (Fotowat et al., 2009; Fotowat and Gabbiani, 2007). As fruit flies and locusts use the angular size of the incoming stimulus, and crabs the angular increment, as a trigger for the decision to take off and initiate an escape from a looming stimulus (Fotowat et al., 2009; Fotowat and Gabbiani, 2007), it seems reasonable that hoverflies could use one of these parameters too. *Eristalis* hoverflies have the neural machinery in place to process the type of stimuli that the incoming insects generate. For example, 50 ms before take-off, the width of the incomer subtended a few degrees on the occupant's eye (Fig. 4B). Female target neurons can track moving targets even smaller than this (Nordström and O'Carroll, 2006). The incomer's angular velocity, 50 ms before take-off, was a few hundred degrees per second (Fig. 4C), which is also within the response range of female target neurons (Nordström and O'Carroll, 2006). However, for all of the four potential visual triggers investigated here, we found a large variation between individual trials (Fig. 4). This suggests that feeding female *Eristalis* might not detect the incomer until late in the interaction because of its attention to feeding, or alternatively that its position was not optimal for detection. Indeed, factors that we did not measure here, such as the energy benefit of the different flowers, the fitness and attention of each individual hoverfly, and the perceived level of threat the incomers posed (Cooper and Frederick, 2007; Ydenberg and Dill, 1986) could explain the large variation across trials (Fig. 4), and also why we observed interactions where the occupant did not leave the flower despite the incoming insect landing on it (Fig. 1A, striped). As an artificial visual stimulus will also induce an escape response in many flies (Card and Dickinson, 2008; Holmqvist and Srinivasan, 1991), visual parameters (Fig. 4) could be manipulated under more controlled conditions in future work using, for example, beads controlled with a rotor (Wardill et al., 2017).

## Supplementary Material

Supplementary information
