# Supplementary material for: Visual approach computation in feeding hoverflies
Source: J Exp Biol. 2018 May 22;221(10):jeb177162. doi: 10.1242/jeb.177162 (PMC5992577; doi:10.1242/jeb.177162)
Supplement: Supplementary information [file jexbio-221-177162-s1.pdf]

Journal of Experimental Biology • Supplementary information

Supplementary Figures

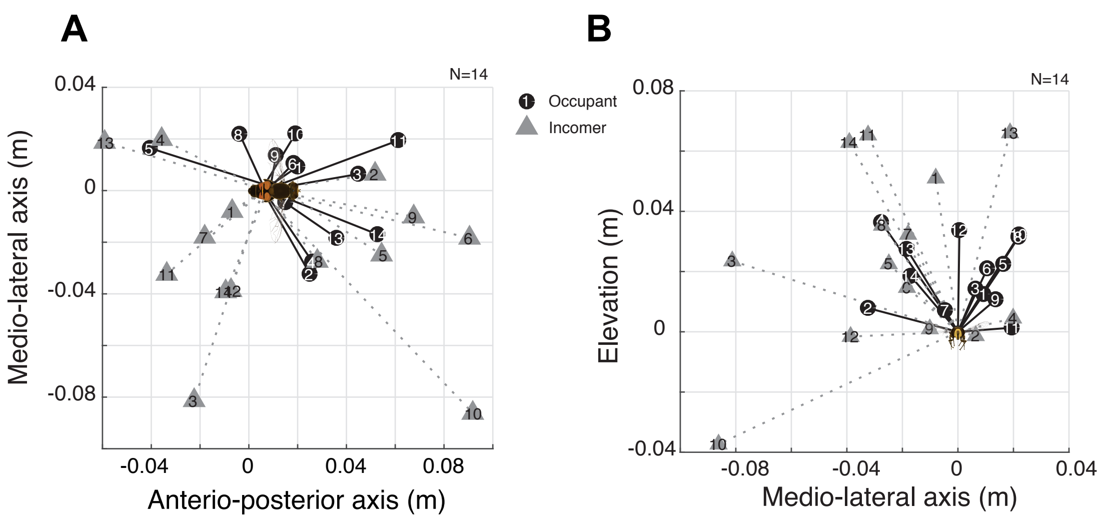

FIGURE S1. THE OCCUPANT PERFORMS AN ESCAPE RESPONSE

A. The same data as in Figure 3C. The graph shows the positions of 14 incomers (female *Eristalis*) 100 ms before occupant take-off (black circles), and the positions of the corresponding occupants (female *Eristalis*) 100 ms after take-off (grey triangles), as viewed from above. The dashed lines join their positions to the position of the occupant at t=0. **B.** The same data as in Figure 3D. The graph shows the positions of 14 incomers (female *Eristalis*) 100 ms before occupant take-off (black circles), and the positions of the corresponding occupants (female *Eristalis*) 100 ms after take-off (grey triangles), as viewed from the front. The dashed lines join their positions to the position of the occupant at t=0.

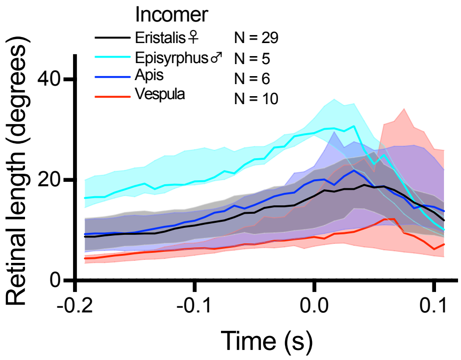

FIGURE S2. VISUAL PARAMETERS – INCOMER LENGTH

The retinal length of the incomer as seen by the occupant. The data have been smoothed using a third order Butterworth filter with a cut-off frequency of 0.25. The color coding indicates incomer species, t=0 is the last frame before the occupant took off from the flower, thick lines show median and shadowing shows the interquartile range. We checked for outliers (Tukey) every 50 ms and excluded any insect which was classified as an outlier for a minimum of 4 time-points.

Supplementary Movies

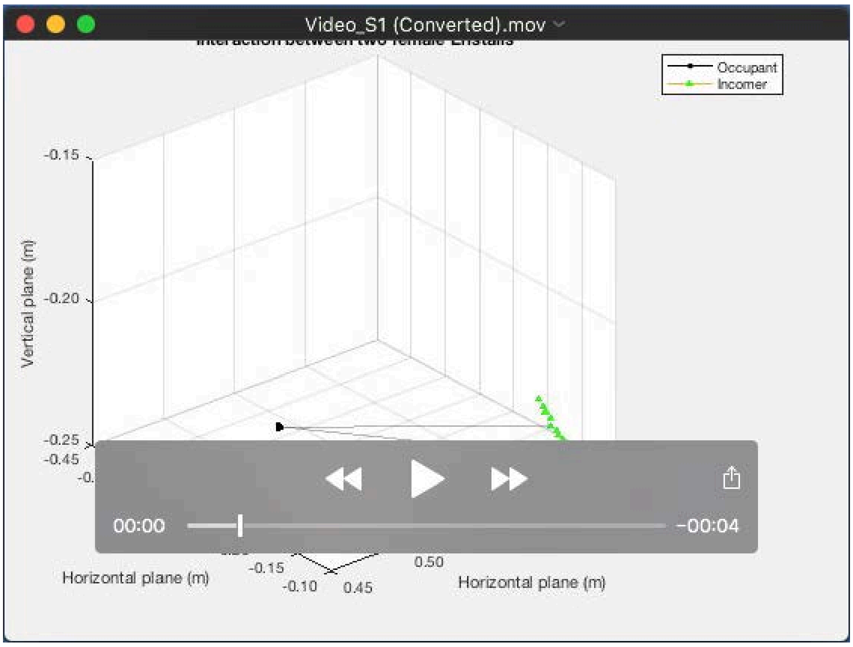

Movie 1. Interaction outcome

Same data as in Figure 1B, showing an example interaction between two female *Eristalis*, occupant displayed with black circles in and incomer in green triangles, where both left the flower. The grey lines connect the position of the occupant and incomer every 20 ms.

Journal of Experimental Biology • Supplementary information
